# Persisting blood–brain barrier disruption following cisplatin treatment in a mouse model of chemotherapy-associated cognitive impairment

**DOI:** 10.1007/s11357-025-01569-x

**Published:** 2025-02-21

**Authors:** Roland Patai, Boglarka Csik, Adam Nyul-Toth, Rafal Gulej, Kiana Vali Kordestan, Siva Sai Chandragiri, Santny Shanmugarama, Stefano Tarantini, Peter Mukli, Anna Ungvari, Andriy Yabluchanskiy, Zoltan Ungvari, Anna Csiszar

**Affiliations:** 1 Vascular Cognitive Impairment, Neurodegeneration and Healthy Brain Aging Program, Department of Neurosurgery, University of Oklahoma Health Sciences Center, Oklahoma City, OK USA; 2 Oklahoma Center for Geroscience and Healthy Brain Aging, University of Oklahoma Health Sciences Center, Oklahoma City, OK USA; 3 International Training Program in Geroscience, Doctoral College/Institute of Preventive Medicine and Public Health, Semmelweis University, Budapest, Hungary; 4 Institute of Preventive Medicine and Public Health, Semmelweis University, Budapest, Hungary; 5 Department of Health Promotion Sciences, College of Public Health, University of Oklahoma Health Sciences Center, Oklahoma City, OK USA; 6 The Peggy and Charles Stephenson Cancer Center, University of Oklahoma Health Sciences Center, Oklahoma City, OK USA; 7 International Training Program in Geroscience, Doctoral College/Institute of Translational Medicine, Semmelweis University, Budapest, Hungary

**Keywords:** Cognitive impairment, Chemobrain, Toxicity, Chemotherapy, Neurovascular unit, Endothelial cell, Cerebral circulation, Neuroinflammation, Cellular senescence, Endothelium, Aging, DNA damage, Side effect, VCI

## Abstract

Chemotherapy-related cognitive impairment, commonly referred to as “chemobrain,” significantly affects cancer survivors’ quality of life, yet its underlying mechanisms remain unclear. Most chemotherapeutic agents cannot cross the blood–brain barrier (BBB), yet they cause central nervous system side effects, suggesting alternative pathways of toxicity. Given that these drugs interact with the cerebrovascular endothelium at their highest concentrations, it is logical to hypothesize that endothelial damage contributes to these effects. Our recent studies demonstrated that paclitaxel-induced cognitive impairment in a mouse model results in a partial BBB disruption and subsequent neuroinflammation, mediated by chemotherapy-induced endothelial senescence. In this pilot study, we used two-photon microscopy to assess BBB permeability in mice receiving a clinically relevant cisplatin regimen, evaluating the leakage of fluorescent dextran tracers of varying molecular weights. Two months post-treatment, cisplatin-treated mice exhibited significantly increased BBB permeability to smaller molecular tracers (40 kDa, 3 kDa, and 0.3 kDa) compared to controls, indicating sustained BBB disruption. These results align with our findings for paclitaxel and suggest that chemotherapy-induced endothelial damage and senescence play a central role in cognitive impairments. Interventions targeting endothelial health could mitigate these long-term effects, improving cognitive outcomes for cancer survivors.

## Introduction

Chemotherapy-related cognitive impairment (CRCI), commonly referred to as “chemobrain,” is a debilitating condition that affects a significant proportion of cancer survivors [1–7]. Characterized by deficits in memory, attention, executive function, and processing speed, CRCI negatively impacts patients’ quality of life and functional independence [8–10]. Despite its prevalence, the mechanisms underlying CRCI remain poorly understood, limiting the development of effective interventions.

Emerging evidence implicates persisting blood–brain barrier (BBB) disruption as a central contributor to CRCI [11]. The BBB, a highly selective barrier formed by tightly connected endothelial cells, astrocytic end-feet, and pericytes, protects the brain from circulating toxins and systemic inflammation [12]. While most chemotherapeutic agents are unable to cross the intact BBB [13], they interact with the cerebrovascular endothelium at their highest concentrations [11].

Our recent studies in a mouse model of paclitaxel-induced chemobrain demonstrated that high concentrations of chemotherapeutic agents can induce DNA damage and cellular senescence in cerebromicrovascular endothelial cells [11]. Senescent endothelial cells undergo profound functional and phenotypic alterations, leading to the disruption of tight junctions and persistent BBB dysfunction [11, 14–19]. This compromise in BBB integrity permits the infiltration of peripheral inflammatory mediators and neurotoxic molecules into the brain, resulting in chronic neuroinflammation and oxidative stress [12]. These processes are hypothesized to play a central role in chemotherapy-associated cognitive decline. Importantly, we established causality by showing that the pharmacological or genetic removal of senescent cells restored BBB integrity and improved cognitive function in paclitaxel-treated mice [11].

Despite these insights, a critical question remains: does persistent BBB disruption occur following treatment with other chemotherapeutic agents? Cisplatin, a platinum-based chemotherapy widely used for solid tumors, is well known for its systemic toxicities and for its ability to induce CRCI [20–29], but it has been less thoroughly investigated for its effects on BBB integrity. To address this gap, we investigated the impact of cisplatin on BBB integrity in a mouse model of CRCI. Using a clinically relevant cisplatin treatment regimen and two-photon microscopy to assess BBB permeability, we evaluated whether cisplatin induces BBB disruption similar to that observed with paclitaxel. By identifying common mechanisms of chemotherapy-induced neurovascular injury, this research provides critical insights into the pathogenesis of CRCI and highlights potential therapeutic targets to mitigate its cognitive effects.

## Materials and methods

### Experimental animals and design

Male C57BL/6 mice were utilized to evaluate the effects of cisplatin on BBB integrity. These mice were chosen as they provide a well-characterized genetic background and are commonly used in preclinical research, ensuring reproducibility and comparability across studies. Mice were housed five per cage in the specific pathogen-free animal facility at the University of Oklahoma Health Sciences Center (OUHSC), maintained on a 12-h light/dark cycle, and provided standard rodent chow and water ad libitum. One week before the initiation of cisplatin treatment, animals were transferred to the conventional animal facility of OUHSC, where they were housed under similar environmental conditions, ensuring continuity in husbandry practices.

The study was designed to evaluate the side effects of cisplatin on BBB integrity independently of the confounding factors associated with cancer itself. Tumors are known to induce systemic effects, including alterations in vascular permeability, immune and inflammatory response, and metabolic status, all of which could independently influence BBB integrity. By focusing on non-tumor-bearing mice, the study isolates the direct impact of cisplatin on the neurovascular unit, allowing for a clearer interpretation of its neurotoxic side effects without interference from cancer-related changes. This approach ensures that observed outcomes can be attributed specifically to the drug’s impact, rather than to a combination of drug and disease-related variables.

#### Treatment protocol

Male C57BL/6 mice (*N* = 10), aged 5–6 months, were randomly assigned to two groups: a cisplatin-treated group (*N* = 3) and a vehicle control group (*N* = 7). Cisplatin (232,120, Sigma-Aldrich, MO, USA) was dissolved in saline and administered intraperitoneally in 2.3 mg/mL dose. The treatment was administered daily for 5 days, followed by a 5-day rest with no injection and then another 5-day injection cycle.

Following the treatment phase, all mice were allowed to recover for 2 months in their original environment to minimize acute effects and ensure that observations reflected persistent rather than transient outcomes of cisplatin exposure. Two months after the conclusion of the cisplatin treatment protocol, BBB integrity was assessed, and mice were subsequently euthanized for tissue collection.

All procedures were conducted in accordance with guidelines approved by the Institutional Animal Care and Use Committee (IACUC) of the University of Oklahoma Health Sciences Center. This ensured that all experiments were performed with the highest standards of animal welfare and ethical responsibility.

### Intravital two-photon microscopy

To assess BBB permeability, mice were equipped with a chronic cranial window and intravital two-photon microscopy-based imaging methods were used as described in our previous publication [30].

#### Chronic cranial window surgery

Our laboratory follows a well-established, approved protocol for cranial window surgery, as outlined in our previous publication [30]. Briefly, mice were anesthetized using isoflurane (2–2.5% for induction, 2% for maintenance; at an air flow rate of ~ 0.8–1 L/min) and positioned on a stereotactic stage under a Zeiss Stemi 2000 stereomicroscope. To protect the eyes, ophthalmic ointment was applied, while the head hair was removed, and the skin was disinfected using povidone-iodine scrub followed by a 70% ethanol wipe to maintain sterility. Full anesthesia was confirmed by the absence of paw and tail reflexes, after which an oval section of skin over the skull was carefully removed with pointed-end scissors, and the skull surface was gently cleaned using a disposable scalpel. A local anesthetic, lidocaine (2% saline solution, Millipore-Sigma, MO, USA), was applied to the skull and allowed to act for a few minutes before the craniotomy procedure. The craniotomy was performed over the somatosensory cortex, located 2–3 mm posterior to the coronal suture and lateral to the sagittal suture, with a diameter of approximately 4 mm. The skull was first thinned in a circular outline using a drill, and once sufficiently thinned, the circular bone segment was removed using fine forceps under a drop of sterile saline. Hemostasis, if needed, was achieved using saline-soaked gelatin sponges (Dengofoam, ref: 600,034, Dengen Dental, WY, USA).

A disinfected 5-mm round glass coverslip (ref: 64–0700, Warner Instruments, MA, USA), cleaned and rinsed with sterile saline, was placed over the craniotomy with a drop of saline underneath. Cyanoacrylate glue was used to secure the coverslip to the surrounding bone, followed by an application of acrylic cement (Dental Cement, ref: 51,459, Stoelting, IL, USA) to reinforce the edges of the glass window and the exposed skull. After the cement hardened, mice were administered extended release buprenorphine (1 mg/kg, subcutaneous injection; Buprenorphine ER, ZooPharm, WY, USA) for pain relief and enrofloxacin (10 mg/kg, subcutaneous injection; Baytril 2.27%, Elanco, IN, USA) for infection prevention.

Post-surgery, mice were transferred to clean cages and monitored until fully awake. Recovery was supported by providing hydrogel (70–01–5022, ClearH2O, ME, USA) and food pellets on the cage floor. Recovery and well-being were observed for up to a week, and veterinarians at OUHSC were consulted if complications arose. Intravital imaging studies commenced 2 to 3 weeks after surgery to ensure complete recovery.

#### Measurement of BBB integrity using intravital two-photon microscopy

Two-photon imaging was performed to assess BBB integrity, as previously described [11, 30]. In each group mice equipped with chronic cranial window underwent isoflurane anesthesia (3% induction, 1.5–2% maintenance, with a flow rate of 0.8–1 L/min) and the head was securely positioned with ear bars into a stereotaxic frame. After adding eye ointment, the setup was moved under an Olympus Fluoview FV1000 two-photon microscope, equipped with a water immersion objective (XLPLN25XWMP, 25 × , 1.05 NA; Olympus, Tokyo, Japan) and an 800-nm laser line for excitation. Emitted light was detected by PMT detectors using three filter sets (420–460, 495–540, and 575–630 nm [30]). Subsequently, 500 kDa FITC-dextran (4 µL per gram of body weight, 2 mg/mL, Millipore-Sigma, MO, USA) was retro-orbitally injected for blood vessel visualization and z-stacks with 5-µm *z*-intervals were captured to establish a baseline, because the BBB is almost impermeable for this size range. On the basis of the microvascular architecture cortical areas for subsequent BBB integrity studies were identified and imaged. First, meningeal vessels were detected and imaging depth of zero was set to them. Cerebral microvessels were examined at ~ 0–200 µm depth. The same laser intensity (5%) and detector sensitivity were used regardless of the depth in the tissue to maintain the reproducibility and comparability with other animals. Images captured immediately after 500 kDa tracer injection served as a background intensity internal control. To detect BBB leakage, FITC-conjugated dextrans of decreasing molecular weights (40-, 3 kDa FITC-dextrans) and 0.3 kDa sodium fluorescein, 4 µL per gram of body weight, 2 mg/mL, respectively (ThermoFisher Scientific, MA, USA) were then retro-orbitally injected sequentially, and 15-min time-*z*-stacks (one *z*-stack per minute) were acquired after each injection, resulting in a hyperstack. For image subtraction analyses, the volumes of interests (VOIs) were selected based on observable microvessels. 508 µm × 508 µm × 50–150 µm (*x*, *y*, *z*) VOI was imaged for *z*-stacks. The corresponding pixel numbers were 512 × 512 × 21; thus, the spatial resolution was approximately 1 µm × 1 µm × 5 µm in *x*, *y*, *z* directions (objective point spread function (PSF) < 1.5 μm) [31].

#### Image analysis

Image analysis was performed using FIJI (ImageJ, version 1.53C; National Institutes of Health, USA) with a custom macro developed based on the method of Nyúl-Tóth et al. [30]. Briefly, TIFF images were organized into time-series *Z*-stacks, which were then 3D corrected to ensure proper alignment. Vascular images recorded after injection of 500 kDa tracer were segmented to create a binary mask, which was subtracted from the 3D-corrected *z*-stack maximum intensity projections of permeability tracer images to exclude intravascular volumes. This process enabled the selective quantification of extravasated tracer intensities at each time point. The final step involved measuring the intensity of the post-subtraction images. Changes in integrated density values were used to calculate relative permeability.

#### Determination of relative apparent solute permeability of cerebral microvessels

To determine relative permeability, the integrated density values of extravascular tracer fluorescence intensities from *z*-stack maximum projections were normalized to baseline intensity (500 kDa FITC-dextran). This normalization minimized distortions caused by imaging errors. The cumulative changes in green fluorescence (*I* [a.u.]) compared to the baseline (*I*_0_ [a.u.]) for each tracer were measured for each experimental animal and were plotted as a function of elapsed time, starting from the initial 500 kDa-only recordings to subsequent imaging with smaller tracers. Measurements were taken at individual time points (~ 15 per tracer), and the corresponding area fractions and normalized intensities were used. The area under the curve (AUC) of these intensity plots was calculated, providing a quantitative comparison of extravasated tracer amounts and allowing the determination of relative apparent solute permeability (*I/I*_0_) differences between subjects.

### Statistical analyses

All data are expressed as mean ± standard error of the mean (SEM). Statistical analyses were performed using GraphPad Prism 9 software (La Jolla, CA, USA). Group comparisons between control and treated animals were conducted using unpaired *t*-tests with Welch’s correction to account for potential differences in variance. A *p*-value of less than 0.05 (*p* < 0.05) was considered statistically significant.

## Results

### Cisplatin promotes persistent BBB disruption

The impact of cisplatin treatment on BBB integrity was assessed using two-photon microscopy to measure the relative permeability of fluorescent tracers in the cerebral microvasculature. FITC-conjugated dextran tracers of varying molecular weights and sodium fluorescein (40 kDa, 3 kDa, and 0.3 kDa) were administered retro-orbitally to the experimental mice, and the changes in fluorescence intensity within the brain parenchyma were monitored over time. In the analysis, the fluorescence intensity changes over time for each tracer were normalized and plotted to generate a dynamic profile of BBB solute permeability. To quantify the relative permeability of the cerebral microcirculation, the area under the curve (AUC) of the normalized intensity-time plots was calculated for each group. This approach provided a robust measure of the extent of BBB disruption.

**Fig. 1:**
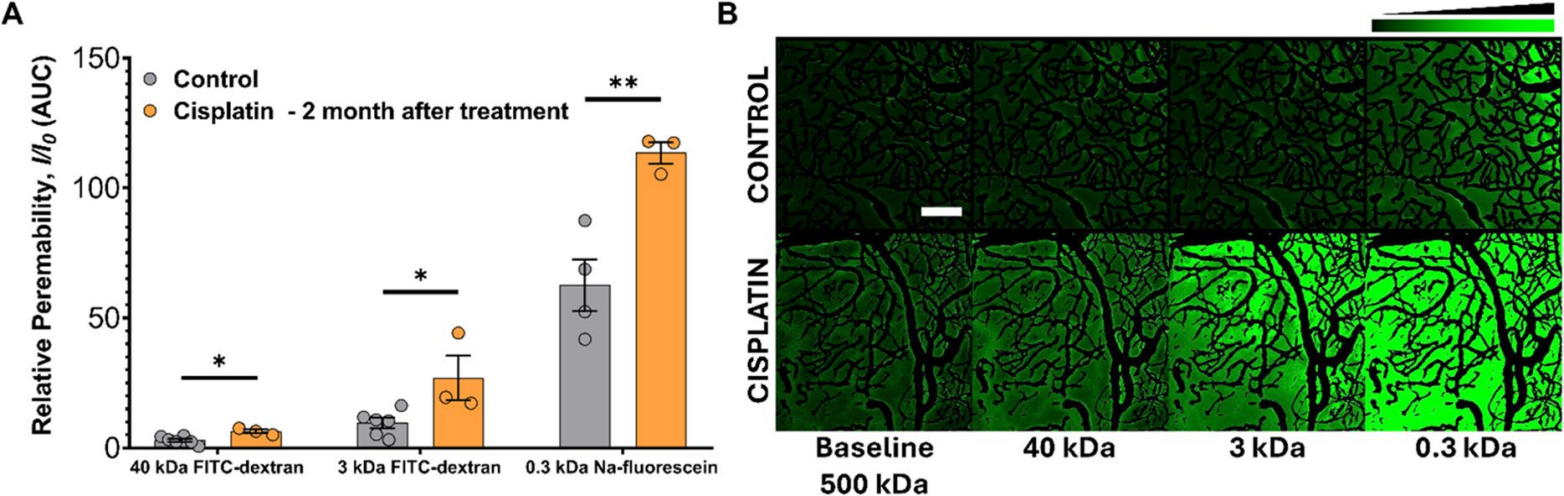
Persistent blood–brain barrier disruption following cisplatin treatment in a mouse model of chemotherapy-associated cognitive impairment. **A** Relative permeability of the blood–brain barrier (BBB) in control and cisplatin-treated mice, measured using FITC-dextran tracers of varying molecular weights (40 kDa, 3 kDa, and 0.3 kDa sodium fluorescein). The data are presented as the area under the curve (AUC) of relative fluorescence intensity changes (*I*/*I*_0_). Significant increases in permeability to all smaller tracers (40 kDa, 3 kDa, and 0.3 kDa) were observed in cisplatin-treated mice 2 months post-treatment compared to controls (*p < 0.05). Data are presented per animal, with each sample representing the average fluorescence intensity values obtained from multiple imaging depths and fields of view per mouse. **B** Representative post-subtraction two-photon microscopy (2PM) images of the cerebral microvasculature from control and cisplatin-treated mice, showing the distribution of FITC-dextran tracers at baseline (500 kDa), 40 kDa, 3 kDa, and 0.3 kDa molecular weights. In cisplatin-treated mice, increased leakage of tracers into the brain parenchyma is evident (enhanced extravascular fluorescent intensity, indicated by color shift from black to green as the scale on the right up shows), indicating persistent BBB disruption 2 months after treatment. Post-subtraction images selectively display extravasated fluorescence signal after subtraction of intravascular fluorescence signal to highlight tracer leakage. Scale: 100 μm

The results revealed that cisplatin-treated mice exhibited significantly increased BBB permeability compared to control animals, across all three tracer sizes (Fig. 1). For the 40 kDa tracer, a moderate but statistically significant increase in extravasation was observed, indicating a compromise in the endothelial barrier to moderately large molecules. The 3 kDa tracer showed a more pronounced level of leakage, suggesting a substantial weakening of tight junctions within the BBB. The 0.3 kDa tracer, the smallest molecular weight tested, displayed the highest level of permeability, underscoring the inability of the BBB to restrict small solutes in cisplatin-treated animals 2 months post-treatment. These findings demonstrate that cisplatin promotes persistent and size-dependent BBB disruption. The degree of permeability increase correlates with the molecular size of the tracer, reflecting a long-term compromise in the barrier’s structural and functional integrity. This persistent disruption of the BBB is likely a key contributor to the development of chemotherapy-induced cognitive impairment, highlighting the long-term neurovascular consequences of cisplatin treatment (Fig. 1).

## Discussion

This study provides compelling evidence that cisplatin treatment induces persistent disruption of BBB integrity, with effects lasting up to 2 months post-treatment. Increased permeability across tracers of varying molecular weights was observed, reflecting both structural and functional compromises in the BBB. These findings indicate long-lasting cerebromicrovascular changes independent of tumor presence, underscoring the neurovascular impact of cisplatin.

Our findings have significant implications for understanding chemotherapy-induced cognitive impairment, or “chemobrain.” Persistent BBB disruption is a plausible mechanism contributing to cognitive deficits observed in both mouse and rat models of cisplatin treatment [20, 21, 23–26, 28, 29]. By allowing neurotoxic molecules and inflammatory mediators to infiltrate the brain, BBB dysfunction may exacerbate neuroinflammation, damage white matter tracts, and impair synaptic connectivity, collectively driving cognitive decline [11, 12, 32, 33]. Given the increasing survival rates of cancer patients, protecting BBB integrity after chemotherapy is crucial to mitigating long-term neurological side effects. Strategies to preserve BBB function could improve quality of life for cancer survivors by reducing the burden of cognitive impairment associated with chemotherapy.

BBB function is determined by three primary pathways: the paracellular pathway, which regulates solute movement between endothelial cells via intercellular junctions (mostly by tight junctions); the transcellular pathway, which involves selective vesicular transport across endothelial cells; and the efflux transport systems. In this study, the observed size-dependent increase in BBB permeability, particularly the pronounced leakage of the smallest tracer (0.3 kDa), strongly suggests that cisplatin primarily disrupts the paracellular pathway. This pathway is regulated by tight junction proteins, and its compromise is consistent with the disruption of endothelial cell junctions known to occur in chemotherapeutic-induced neurovascular damage [34–37]. Adverse effects of cisplatin on tight junction protein expression and endothelial barrier function have been well-documented in vitro using cultured endothelial cells [38]. These studies reveal that cisplatin disrupts the expression and localization of key tight junction proteins, leading to compromised barrier integrity and increased permeability [38]. These results also align with prior studies demonstrating chemotherapeutic-induced microvascular damage [11, 35–37], further supporting the hypothesis that cisplatin impairs BBB function via direct action on the neurovascular unit. Cisplatin-induced disruption of microvascular barrier function through mechanisms such as tight junction loosening has also been implicated in ototoxicity, with studies demonstrating increased vascular permeability in the stria vascularis leading to blood-labyrinth barrier breakdown [37]. Interestingly, cisplatin-induced Sertoli cell toxicity has also been linked to disruptions in junctional proteins, with studies showing that cisplatin reduces the expression and induces aberrant intracellular localization of key tight junction proteins such as occludin and ZO-1, as well as anchoring and gap junction components like N-cadherin and connexin 43, thereby impairing the intercellular junctions essential for spermatogenesis [39]. The impact of cisplatin on tight junction proteins, such as ZO-1 and occludin [40–42], may also involve disruptions to key signaling pathways and post-translational modifications that regulate their stability and localization. For instance, cisplatin has been shown to induce oxidative stress and activate inflammatory pathways [42], such as the NF-κB signaling cascade [43, 44], which can downregulate tight junction proteins or promote their mislocalization through phosphorylation-dependent mechanisms [45, 46]. Additionally, cisplatin may increase the activity of matrix metalloproteinases (MMPs), which degrade extracellular matrix components and destabilize junctional complexes [47, 48]. Post-translational modifications, such as ubiquitination and proteasomal degradation, could also play a role in the loss of tight junction integrity. Furthermore, phosphorylation of tight junction proteins at specific sites may alter their interactions with the cytoskeleton, promoting disassembly of junctional complexes and increasing endothelial permeability. Exploring these molecular alterations could provide deeper insights into how cisplatin compromises BBB function and identify potential therapeutic targets to mitigate its neurovascular toxicity. The use of non-tumor-bearing mice in this study was a critical design choice to eliminate cancer-related confounders such as systemic inflammation, metabolic alterations, or tumor-secreted factors that could independently affect BBB integrity. By isolating the effects of cisplatin, this approach allowed for a clearer understanding of its direct neurovascular toxicity, unclouded by the complex interplay between cancer and treatment.

Cisplatin, like many other chemotherapeutic agents, exerts its cytotoxic effects primarily through inducing DNA damage, which can trigger cellular senescence [49–58]. Endothelial senescence, a key factor in BBB disruption [14, 15, 17, 19, 59, 60], has been well-documented in the context of other chemotherapeutic agents, such as paclitaxel [11], doxorubicin [61], and 5-fluorouracil [62]. These agents cause DNA damage in endothelial cells, leading to an irreversible cell cycle arrest [33]. This process is characterized by profound alterations in endothelial cell function and phenotype, including a loss of endothelial integrity, increased permeability, changes in the extracellular matrix and expression of a wide range of inflammatory factors, all of which contribute to BBB dysfunction [15, 63–68]. Cisplatin-induced DNA damage activates the DNA damage response pathways, including the p53 signaling axis, driving cell cycle arrest and ultimately promoting endothelial senescence [33, 69]. Senescent endothelial cells exhibit distinct gene expression profiles and heightened secretion of pro-inflammatory cytokines and matrix metalloproteinases, which not only disrupt their own function but also influence neighboring endothelial cells, exacerbating and perpetuating blood–brain barrier impairment [15–17, 19, 70, 71].

In addition to promoting DNA damage mediated endothelial senescence, cisplatin treatment amplifies cellular oxidative stress [38, 72, 73]. Reactive oxygen species (ROS) generated by cisplatin can damage cellular structures and exacerbate the senescence phenotype. The accumulation of oxidative damage impairs the function of endothelial cells and other components of the neurovascular unit (NVU), such as astrocytes and pericytes, which are crucial for maintaining BBB integrity. This increased oxidative stress further destabilizes the endothelial barrier [38, 72, 74–76], rendering it more permeable to harmful substances, including inflammatory cytokines and neurotoxic molecules.

Chronic low-grade inflammation is another consequence of cisplatin treatment [44, 73, 77–79], which may also contribute to persistent BBB dysfunction [80]. The senescent endothelial cells, along with other damaged cells in the NVU, secrete pro-inflammatory factors, often referred to as the senescence-associated secretory phenotype (SASP) [64, 67, 68, 70]. The SASP promotes inflammation, contributes to the induction of senescence in neighboring cells (paracrine senescence) [67, 81] and exacerbates BBB disruption. This chronic inflammation can persist well beyond the initial chemotherapeutic insult, further compromising the structural and functional integrity of the blood–brain barrier [11].

While this study primarily characterizes the long-term effects of cisplatin on BBB integrity, the observed BBB disruption highlights an urgent need for therapeutic interventions to mitigate neurovascular toxicity. BBB dysfunction is a key contributor to chemotherapy-induced cognitive impairment, and strategies to protect and restore the integrity of the neurovascular unit could have significant clinical implications for cancer patients undergoing chemotherapy. Several potential therapeutic strategies warrant further investigation, including antioxidants to counteract oxidative stress, senolytic agents to eliminate senescent cells, targeting the SASP, and tight junction stabilization. Cisplatin treatment is known to induce oxidative stress, which damages endothelial cells, disrupts tight junction proteins, and contributes to BBB dysfunction. Antioxidants may help neutralize reactive oxygen species (ROS), thereby preventing oxidative damage and preserving BBB integrity. Preclinical studies have demonstrated that antioxidant therapy can protect endothelial function in various neurovascular injury models, suggesting its potential application in chemotherapy-induced BBB disruption. Endothelial senescence has emerged as a key contributor to BBB dysfunction [11]. Senolytic agents, such as BCL-2 inhibitors [11], have shown promise in selectively eliminating senescent cells and improving vascular health in mouse models of chemobrain. Targeting endothelial senescence may also help restore BBB integrity and reduce neuroinflammation following cisplatin treatment. In addition to eliminating senescent cells, directly modulating the SASP could help reduce the chronic inflammatory environment that drives BBB dysfunction. SASP-targeting strategies, including JAK/STAT inhibitors and IL-6/IL-1β blockade, could suppress the release of pro-inflammatory cytokines and matrix metalloproteinases that contribute to barrier breakdown. By dampening SASP-driven inflammation, these approaches may help preserve BBB function in chemotherapy-exposed patients. Tight junction disruption is a hallmark of cisplatin-induced BBB permeability changes. Pharmacological agents that reinforce tight junction protein expression or prevent their degradation, such as glucocorticoids, could help maintain BBB integrity. Targeting intracellular signaling pathways that regulate tight junction dynamics may offer additional therapeutic avenues to reduce vascular permeability. Future studies should explore these therapeutic strategies in preclinical and clinical settings to determine their efficacy in mitigating cisplatin-induced BBB disruption. A combination approach targeting oxidative stress, endothelial senescence, and inflammatory signaling may be particularly effective in preserving BBB function and reducing the risk of chemotherapy-induced cognitive impairment. Ultimately, identifying neurovascular protective interventions could improve the quality of life and neurological outcomes for cancer survivors undergoing cisplatin-based chemotherapy.

This pilot study has several limitations that warrant discussion. First, the exclusive use of male mice precludes any analysis of potential sex-specific differences in BBB disruption or recovery. Further studies involving female mice are necessary to explore whether hormonal differences influence the vascular effects of cisplatin. Second, the study did not evaluate dose dependency. Future studies should explore dose–response relationships to identify the thresholds at which BBB integrity is compromised and to determine whether lower doses might mitigate neurovascular toxicity while maintaining therapeutic efficacy. Such studies could provide clinically relevant guidance for optimizing chemotherapy regimens with reduced neurotoxic side effects. Another limitation of this study is the lack of direct behavioral assessment to correlate BBB disruption with cognitive deficits. While previous studies have linked chemotherapy-induced BBB dysfunction to cognitive impairment, future research should incorporate behavioral testing to establish a direct relationship between neurovascular damage and cognitive performance in cisplatin-treated animals.

Future research should focus on developing protective strategies to maintain BBB integrity after chemotherapy. While this study utilized non-tumor-bearing mice to isolate the direct effects of cisplatin on BBB integrity, future studies should assess BBB function in tumor-bearing models to determine whether the presence of cancer exacerbates chemotherapy-induced neurovascular damage. Tumor-induced inflammation and metabolic alterations could interact with cisplatin toxicity, potentially leading to even greater BBB dysfunction. Future studies should also incorporate advanced molecular approaches, such as single-cell RNA sequencing, to further characterize the cellular heterogeneity and transcriptomic changes in the neurovascular unit following cisplatin treatment. This approach could provide mechanistic insights into endothelial dysfunction, tight junction protein dysregulation, and inflammatory signaling pathways contributing to persistent BBB disruption.

In conclusion, this study underscores the potential role of BBB dysfunction in the pathogenesis of cisplatin chemotherapy-induced cognitive impairment, offering a translational bridge to better understand its neurovascular underpinnings. The persistent BBB disruption observed in this study suggests the need for targeted interventions to mitigate the neurovascular toxicity of cisplatin. Antioxidants, which counteract oxidative stress [29], could be a promising strategy to protect BBB integrity by preventing ROS-induced damage to tight junction proteins and endothelial cells. Additionally, senolytic agents, which eliminate senescent cells, may help restore BBB functionality by reducing the deleterious effects of the SASP [11]. Targeting SASP directly, for instance, through anti-inflammatory therapies or matrix metalloproteinase inhibitors, could further alleviate chronic inflammation and preserve the BBB. Other approaches, such as reinforcing tight junction stability using pharmacological agents may also hold therapeutic potential. These therapeutic strategies hold the potential to minimize neurovascular side effects, mitigate the risk of chemobrain, and significantly improve the quality of life and long-term outcomes for cancer survivors. Future research should prioritize the exploration and validation of these interventions, paving the way for effective clinical applications to protect the BBB after chemotherapy.
